# Facile Synthesis of Pyridyl Rosamines as Potential Photosensitizers

**DOI:** 10.3390/ijms26041482

**Published:** 2025-02-10

**Authors:** Éva Bakos, Henrietta Ágoston, Rebeka Ignácz, Attila Hunyadi, Miklós Poór, János Erostyák, Zoltán Kele, Csilla Özvegy-Laczka, Erzsébet Mernyák

**Affiliations:** 1 Drug Resistance Research Group, Institute of Molecular Life Sciences, Research Centre for Natural Sciences, Hungarian Research Network, Magyar Tudósok Körútja 2, H-1117 Budapest, Hungarylaczka.csilla@ttk.hu (C.Ö.-L.); 2 Department of Molecular and Analytical Chemistry, University of Szeged, Dóm tér 8, H-6720 Szeged, Hungary; hencsike12@gmail.com (H.Á.); ignaczrebi2000@gmail.com (R.I.); 3 Department of Pharmacognosy, University of Szeged, Eötvös u. 6, H-6720 Szeged, Hungary; hunyadi.attila@szte.hu; 4 Biologically Active Natural Products Research Group, Hungarian Research Network, University of Szeged, Eötvös u. 6, H-6720 Szeged, Hungary; 5 Department of Laboratory Medicine, Medical School, University of Pécs, Ifjúság útja 13, H-7624 Pécs, Hungary; poor.miklos@pte.hu; 6 Molecular Medicine Research Group, János Szentágothai Research Centre, University of Pécs, Ifjúság útja 20, H-7624 Pécs, Hungary; 7 Department of Experimental Physics, Faculty of Sciences, University of Pécs, Ifjúság útja 6, H-7624 Pécs, Hungary; erostyak@fizika.ttk.pte.hu; 8 Molecular Biophysics Research Group, János Szentágothai Research Centre, University of Pécs, Ifjúság útja 20, H-7624 Pécs, Hungary; 9 Department of Medicinal Chemistry, University of Szeged, Dóm tér 8, H-6720 Szeged, Hungary; kele.zoltan@med.u-szeged.hu

**Keywords:** rosamine, microwave-assisted, fluorophore, mitocan, photodynamic therapy

## Abstract

Rosamines represent one of the most promising groups of xanthene dyes. Their excellent photophysical properties allow their widespread application. Their use as photosensitizers in photodynamic therapy has recently gained considerable attention. Here, we report the facile, effective, microwave-assisted synthesis of rosamine dyes. Pyridylbenzaldehyde derivatives were reacted with 1,3-dialkylaminophenols or 8-hydroxyjulolidine in toluene without any additive. The resulting pyridyl rosamines were investigated for their cytotoxic effect against the A431 human epidermoid carcinoma cell line to estimate their potential as photosensitizers. The compounds displayed light-induced toxicity in the submicromolar or occasionally in the low nanomolar range. One of the julolidine-based derivatives exhibited a phototoxic index above 100, indicating an increase in light-induced cytotoxic efficacy of two orders of magnitude compared to its negligible dark toxicity. This compound is a particularly promising candidate for the development of novel pyridyl-rosamine-based photosensitizers for photodynamic therapy of skin cancer.

## 1. Introduction

**Figure 1:**
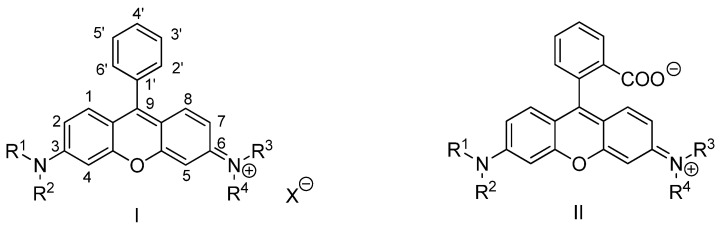
Structure of rosamine (**I**) and rhodamine (**II**) scaffold.

Rosamines (I), together with rhodamines (II) and fluoresceins, belong to the group of xanthene dyes (Figure 1) [1,2]. The outstanding photophysical properties of these xanthene dyes facilitate their widespread application. For example, rhodamine fluorophores (II) have been applied as fluorescent probes and sensors [3,4,5,6]. Owing to the pH-sensitive fluorescent properties of their spirolactam derivatives, they might act as pH sensors [7,8,9]. Nevertheless, the carboxyphenyl side chain present in position 9 of the xanthene core might complicate their synthesis, in particular, regarding regioselectivity. Rosamines (I), lacking the carboxylic group, are relatively little studied, despite the fact that they possess similar excellent photophysical properties. High molar absorption coefficients, high fluorescence quantum yields in the visible or red spectral region as well as a relatively high photostability allow the applications of both dyes in several fields.

Lipophilic cations such as rhodamines (II) or rosamines (I) passively penetrate the plasma membrane, and they accumulate selectively in the inner mitochondrial membrane of cancer cells. The relatively higher net negative mitochondrial transmembrane potential of cancer cells in comparison with that of normal cells allows the delocalized lipophilic cations, such as rosamines, to selectively accumulate in the inner mitochondrial membrane of cancer cells. The reduction in the mitochondrial membrane potential might result in reactive oxygen species (ROS) generation followed by apoptosis. Being selective in targeting cancer cells, these mitochondria-targeting drugs, the so-called mitocans, might represent an important class of anticancer compounds. The literature reveals certain rhodamine or rosamine derivatives as potent mitocans; however, their cancer selectivity still remains improvable. Relatively few investigations, describing the cell line-selectivity of rosamines in vitro, have been carried out [10]. A rhodamine derivative, namely Rhodamine 123, has been tested in a clinical trial against prostate cancer. Note, however, that its dosage was not tolerable, and this led to inefficient treatment [11]. Nowadays, substantial effort has been put in the development of mitocans as photosensitizers (PSs) applicable in photodynamic therapy (PDT). PDT is a photochemical-based treatment strategy, which uses light combined with a light-activated chemical compound as a PS [12]. The most favorable properties of this therapeutic approach involve its noninvasive character, few adverse or side effects, short treatment time, repeatability, and local applicability [13,14]. π-extended thio- and seleno-rhodamines proved to be effective photosensitizers in vitro toward Colo-26 cancer cells [15]. Liu et al. combined a rosamine dye with luminescent transition-metal centers [16]. These hybrids possess high in vitro and in vivo phototoxicity with low dark toxicity. Very recently, a class of bio-orthogonally activatable PSs based on Se-rhodamine core appeared [17]. The π-extended seleno-rhodamine azides possessed neither dark nor phototoxicity. However, their phosphine reduction resulted in efficient phototoxic amino PSs against HeLa cells. Accordingly, rhodamine and rosamine scaffolds might serve as a promising basis for the design of effective PSs for PDT of cancer. Due to their favorable photophysical and biological properties, these dyes represent an important class of phototheranostic (PT) agents. Their multiple potential, namely their ability to simultaneously provide diagnostic signals and light-induced therapeutic effects, assigns them great value in medicine.

**Scheme 1:**
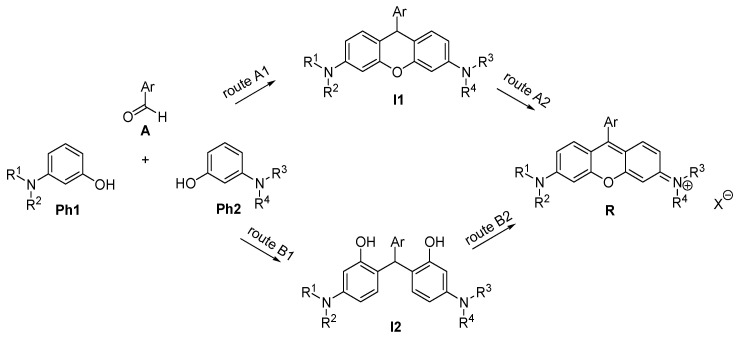
Route A1: propionic acid or water, *p*-TsOH, reflux or microwave irradiation; route A2: oxidation with chloranil in toluene; route B1: propionic acid or water, *p*-TsOH, ohmic heating; oxidation with chloranil; route B2: Lewis acids in toluene.

In order to develop potent PT agents, their synthetic availability is crucial. Although certain rhodamine dyes are commercially available, they are often quite expensive, and their postfunctionalization is highly limited. Introduction of the desired functional groups is generally solved by utilization of starting compounds already bearing the appropriate functions. However, the high reaction temperatures and the acid solvents or catalysts do not allow the use of starting compounds, which are sensitive to high-temperature and acidic conditions. These inconveniences might be avoided by the introduction of protecting groups, but this strategy increases the number of reaction steps, thereby decreasing the overall yield. There exist three main approaches for the synthesis of rosamine dyes [18,19,20]. The first route is based on the condensation of an aromatic aldehyde with *meta*-dialkylaminophenol derivatives and subsequent oxidation (route A1 and route A2, Scheme 1) [18]. The literature describes conventional heating or microwave (MW) irradiation methodology for accomplishing the condensation. Propionic acid and water are usually used as solvents, and *p*-TsOH as an acid catalyst. A microwave-assisted protocol in propionic acid solvent, using *p*-TsOH as an acid catalyst, led to the desired rosamine dyes in a maximum yield of 51%. However, reactions in water afforded the dyes in 68% yield. In microwave-irradiation protocols, the amount of acids is limited, since side reactions may occur. Another weakness of the first route originates from the oxidation step, which might result in substantial yield loss. The second route is the ohmic heating approach to give triarylmethane intermediates, followed by oxidative catalytic cyclization (route B1 and route B2, Scheme 1) [18]. The formation of the intermediates could be achieved by ohmic heating with low yields. Importantly, however, efficiency could be improved by using 0.8 equiv. of *p*-TsOH as an acid catalyst. Besides the triarylmethane intermediate, a very small amount of rosamine (under 10%) was detected in the reaction mixture. Subsequent oxidation was carried out using certain Lewis acids. Heating trialrylmethane in propionic acid at 100 °C promoted the formation of the rosamine in high yields. However, the overall yields obtained in this two-step protocol still needed to be improved. Jiao et al. described the microwave-assisted preparation of a rosamine derivative without using any solvent or acid [21]. 7-Hydroxy-*N*-methyl-2,2,4-trimethy-l,2-dihydroquinoline was reacted with 4-bromobenzaldehyde. This protocol allowed the synthesis of the desired dye in 53% overall yield. Accordingly, reactions of 3-aminophenol derivatives and 8-hydroxyjulolidine were performed in 60% H_2_SO_4_ under MW heating.

To the best of our knowledge, there is no example in the literature for the effective synthetic protocol of rosamine dyes (I) based on aldehyde–phenol condensation strategy, starting from 3-dialkylaminophenols or 8-hydroxyjulolidine, without the use of any acid, as a catalyst or as a solvent.

**Scheme 2:**
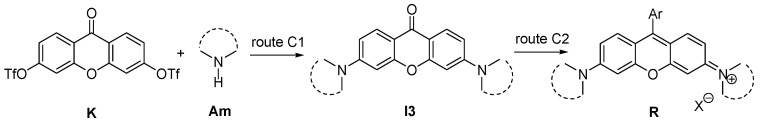
Route C1: DMSO, conventional heating; route C2: nucleophilic addition with Grignard or organolithium reagents.

The third route relies on S_N_Ar reactions of a readily accessible xanthone ditriflate with cyclic amines [19,20]. A subsequent addition reaction with organolithium or Grignard reagents, then protonation, followed by dehydration resulted in rosamine dyes (route C1 and C2, Scheme 2). This approach was achieved both in the solution and in the solid phase. Nevertheless, certain preparative difficulties arose concerning these approaches, too.

9-Pyridyl rosamines represent a promising group of compounds in several aspects [22,23]. Due to its high dipole moment, the pyridine ring has higher electronegativity than the phenyl group lacking nitrogen. Accordingly, optical properties of pyridyl rosamines differ substantially from those of their phenyl counterparts. The position of the nitrogen in the heterocyclic ring at C-9 slightly influences the absorption and emission properties of the compounds. The pyridinium salt formed via alkylation of the pyridine nitrogen is often found in natural products and other biologically active compounds. Cationization results in a marked bathochromic shift and a fluorescence quenching via the d-PET mechanism. *N*-substituted pyridium rosamine derivatives have recently been reported as mitochondrial-targeting agents [22]. Lioret et al. recently described a novel class of enzyme-activatable phototeranostics based on hetero-rosamine scaffold [23]. The *meso*-4-pyridyl moiety present in the S- or Se-rosamine core assigns them photoactivity, which relies on the caging/decaging status of the *meso*-heterocycle. The recognition moiety was introduced by directed *N*-quaternarization with special substituents. The conjugates proved to be applicable in combined diagnosis and PDT.

Owing to the great potential in rosamine-based PS or PT agents, there is a high demand for elaboration of improved synthetic strategies. Evaluation of their dark- and light-induced cytotoxicity against various cancerous and normal cell lines would also be of great value. Accordingly, here, we have planned to develop an efficient but simple synthetic methodology to obtain 9-pyridyl rosamines. We intended to avoid utilization of any additives or catalysts. The improvement of the oxidation step was also in our focus. We aimed to characterize the photophysical properties of the rosamine dyes. Additionally, investigation of the synthesized rosamines as potential PSs against A431 cancer cell lines was planned in vitro.

## 2. Results and Discussion

### 2.1. Synthesis of Rosamines (**8**–**16**)

**Scheme 3:**
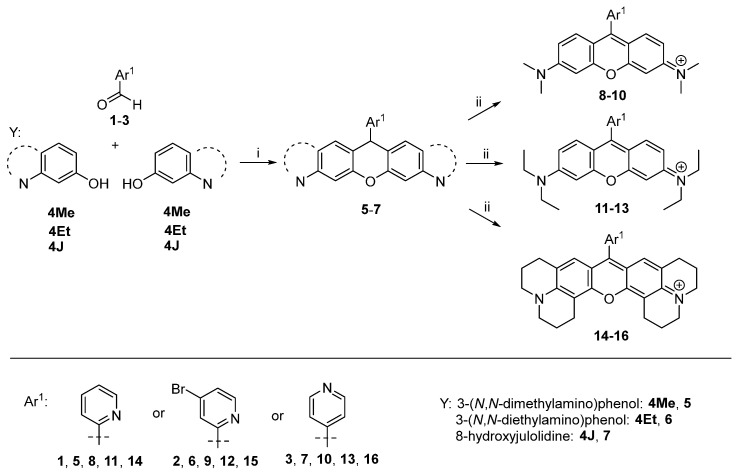
Synthesis of a nine-membered rosamine library via aldehyde−pyrrole condensation—oxidation strategy. Reagents and conditions: (i) phenol (2.0 equiv.), aldehyde (1.0 equiv.), toluene, 150 °C, MW, 30 min; (ii) 10% chloranil (1.8 equiv.) in dichloromethane/methanol = 1/1, 0−5 °C, 30 min.

The aldehyde–pyrrole condensation strategy was chosen to produce rosamine derivatives efficiently. We have designed and synthesized a nine-member compound library consisting of combinations of three different aminophenol (**4Me**, **4Et**, **4J**) and pyridine-carbaldehyde (**1**–**3**) units (Scheme 3). The heteroaromatic aldehydes (**1**–**3**) utilized as starting compounds differ in the position of the nitrogen and the presence of a bromo substituent. We believe that the high but different dipole moment of the heterocyclic ring derived from the aldehydes might have substantial impact on the photophysical properties of the dyes. Rosamines bearing pyridyl moieties allow postfunctionalization not only on their xanthene core, but at their nitrogen atom as well. Due to their bromoaryl character, bromopyridyl derivatives can be promising starting compounds for cross-coupling reactions catalyzed by transition metals. Concerning the phenol counterpart, two *meta*-dialkylaminophenol (**4Me**, **4Et**) derivatives or a julolidine-based phenol (**4J**) were used. The latter can be particularly beneficial due to the induced bathochromic shift. Condensation of the aldehydes (**1**–**3**) with two equivalents of phenol derivatives (**4Me**, **4Et**, **4J**) was performed by heating the reaction mixture in a microwave reactor in toluene solvent, without the addition of any catalyst. The best results were obtained by 30 min irradiation at 150 °C. The starting compounds were completely consumed, and the desired compounds were formed as the major products (**5**–**7**). The nature of neither the aldehyde nor the phenol reactant had a significant effect on the outcome of the condensations. Higher temperatures and/or longer reaction times resulted presumably in the decomposition of the starting compounds and/or products, as shown by TLC. No in-depth analysis of the by-products has been carried out, but our investigations, among others, indicated the partial dealkylation of the aminophenol building blocks. Intermediates **5**–**7** bearing the C(sp^3^)-9 carbon atoms are air-sensitive, as they are already partially oxidized prior to oxidation. The syntheses were continued in two ways. According to the first method, the intermediates (**5**–**7**) were subjected to crude column chromatography, and the resulting mixture of the two products differing in the hybrid state of C-9 was reacted with the oxidizing agent. The other method was oxidation without purification of the intermediates (**5**–**7**). An overall yield increase of around 10% can be achieved with the first method. The choice of reagent and reaction conditions used for oxidation is the critical point in the synthesis, as most of the material loss typically occurs in this phase. The literature suggests the utilization of benzoquinone-type oxidants for this purpose to carry out the process at room temperature or by mild heating usually in dichloromethane as a solvent [18,19,20]. In contrast, we dissolved the intermediates in dichloromethane and kept the reaction mixture at room temperature or cooled it to 0–5 °C (Table 1). The oxidation was tested with three different oxidizing agents, namely 1,4-benzoquinone, DDQ (2,3-dichloro-5,6-dicyano-1,4-benzoquinone), and chloranil (tetrachloro-*p*-benzoquinone). The addition of the oxidizing agent was performed in two ways, by adding the solid reagent in one portion or in 10% solution in dichloromethane/methanol (1/1). The highest yields were obtained by slow addition of a chloranil solution at 0–5 °C reaction temperature (Table 1, entry 7). The large differences in the effectiveness of the tested methods highlight the importance of the thorough investigation of both the condensation and oxidation steps.

The structures of the newly synthesized rosamine derivatives (**8**–**16**) were evaluated from their ^1^H and ^13^C NMR spectra.

### 2.2. Photophysical Characterization of Rosamines (**8**–**16**)

First, the absorption and emission spectra of **8**–**16** were examined in DMSO (Figure S1), where the absorption wavelength maxima of rosamine derivatives were in the 570–599 nm range, and the emission wavelength maxima were between 595 and 623 nm. In DMSO, compounds **11** and **12** showed the highest absorbance, while **13** exerted the strongest emission signal, followed by **16** and **11**.

**Figure 2:**
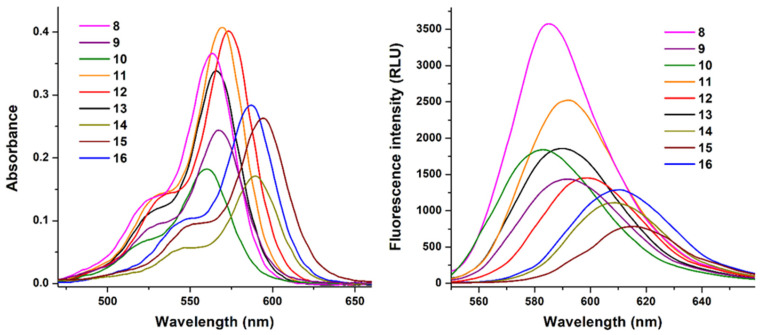
Representative UV-Vis absorption (**left**) and fluorescence emission (**right**) spectra of **8**–**16** (4 μM each) in PBS (pH 7.4; see λ_abs_ values in Table 2).

Thereafter, the spectroscopic properties of **8**–**16** were tested in an aqueous environment (PBS buffer), where the blue shifts of both absorption (4–11 nm) and emission (5–13 nm) wavelength maxima were noticed. The representative UV-Vis absorption and the fluorescence emission spectra (in PBS, pH 7.4) of **8**–**16** are shown in Figure 2, while the spectroscopic data of rosamine derivatives examined are listed in Table 2. In PBS, absorption maxima of the compounds tested were between 559 and 594 nm. Compounds **11** and **12** showed the highest absorbance values, while the lowest data were measured with **14** and **10**. The strongest emission signal was exerted by **8** followed by **11**, and derivative **15** showed the lowest fluorescence intensity. We noticed the emission wavelength maxima of **8**–**16** between 583 and 617 nm. It can be stated that no strong correlation exists between the structure of rosamines and their fluorescence intensities. Slight differences in the absorption or emission maxima appear concerning the dialkylaminophenol derivatives with pyridyl rosamine units bearing a nitrogen at different positions. Nevertheless, the emission maxima of the julolidine derivatives (**14**–**16**), as expected, shifted to above 600 nm. Our results are in line with those measured by Leite et al. for *N*-diethyl derivatives bearing three different pyridyl units [18]. It should be stressed, however, that they did not investigate the effect of photophysical properties, when replacing the aminophenol building blocks by julolidine and the presence of bromine. They found that the position of a heteroatom in the pyridyl ring does not affect the fluorescence quantum yields. In contrast, the polarity of the solvent resulted in a significant change: the more polar the solvent in which the quantum yield was measured, the lower the values obtained. Taking all this into account, we selected two rosamines (**12** and **16**) that seemed to be biologically relevant and determined their absolute fluorescence quantum yields. The following values were obtained: 15.3% (±0.2%) for **12** and 18.9% (±0.5%) for **16**. These data correspond to those described in the literature measured in water (16–19%) [23].

### 2.3. Evaluation of In Vitro Dark- and Light-Induced Toxicity of Rosamines (**8**–**16**)

**Figure 3:**
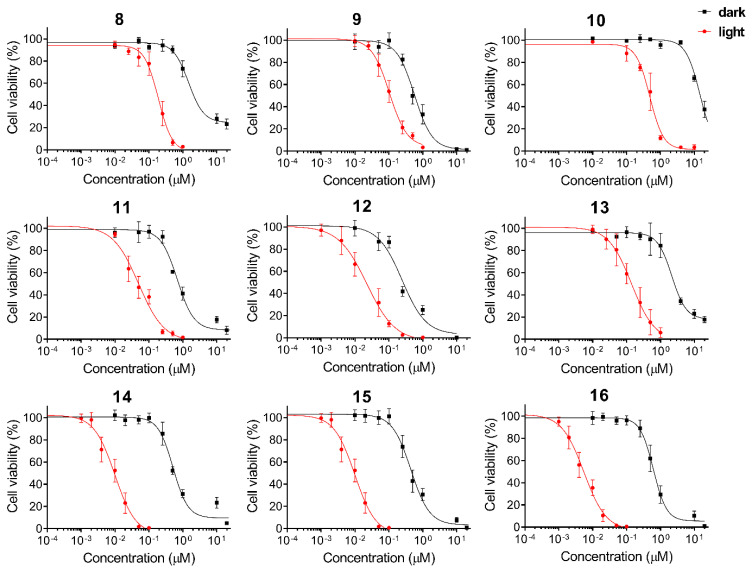
Effect of pyridyl rosamines on the viability of A431 cells with and without light irradiation. A431 cells were incubated with or without the test compounds for 24 h. After incubation, the compounds were replaced with fresh cell culture medium, and the cells were irradiated for 10 min using an LED source at either 550 nm (for experiments **8**–**13**) or 605 nm (for experiments **14**–**16**). The cells were then cultured in the dark. Cell viability was assessed 48 h post irradiation using the PrestoBlue assay. In parallel, dark-induced toxicity was evaluated for the compounds without light exposure. Data are presented as the mean ± SD (*n* = 3).

The phototoxic properties of pyridyl rosamines (**8**–**16**) were studied against A431 human epidermoid carcinoma cells, a well-established model in toxicology and cancer research. Cells were incubated either with or without the compounds for 24 h, after which the compounds were replaced with fresh cell culture medium, and the cells were irradiated with an LED source of 550 nm (**8**–**13**) or 605 nm (**14**–**16**) for 10 min and further cultured in dark. Cell viability was determined 48 h post irradiation. The dark toxicity of the test compounds was also evaluated in parallel without light irradiation. IC_50_ values calculated for dark toxicity ranged from submicromolar to about 15 micromolar concentrations (Figure 3 and Table 3). It was expected that these rosamines would display a certain dark toxicity, due to their potential mitocan properties [10]. We were particularly interested in whether there was a significant difference between the dark- and light-induced toxicity, and if so, with what selectivity. To our great delight, the light-induced toxicities were found to be significantly higher for all test compounds. The phototoxic index (PI), defined as the ratio of dark to light IC_50_ values, serves as a measure of the light-induced cytotoxic efficacy of a PS relative to its dark-induced cytotoxicity. We observed PS values between 5 and 127.

As summarized in Table 3, julolidine derivatives **14**–**16** demonstrated the highest light-induced toxicity, with IC_50_ values in the low nanomolar range. Two 2-pyridyl derivatives (**14** and **15**) displayed similar IC_50_ and PI (near 50) values, regardless of the presence of the bromine atom. However, the introduction of the *meso*-(4-pyridyl) moiety (compound **16**) resulted in a slight decrease in dark- but a marked increase in light-induced toxicity. This compound was the most potent, displaying the best photocytotoxic activity with IC_50_ of 6 nM upon light irradiation, with an outstanding PI of 127. The two *meta*-diethylamino compounds (**11** and **12**) displayed somewhat higher light-induced IC_50_ values, but still in the nanomolar range (53 and 20 nM, respectively). Their dimethylamino counterparts (**8** and **9**) and 4-pyridyl derivative **13** proved to be less potent with low submicromolar IC_50_ values, while compound 10 had the highest IC_50_ (572 nM) under light irradiation. From comparison of the results by structurally related dialkylphenol triads (**8**–**10** or **11**–**13**), interesting structure–effect relationships emerge. The bromo derivatives (**9** or **12**) are the most potent, and the 4-pyridyl derivatives (**10** or **13**) are the least potent in terms of both dark- and light-induced toxicity IC_50_ values. However, this correlation is not true for the third triad (**14**–**16**). Certain recent literature data suggest that introduction of the julolidine moiety into the organic dyes significantly modifies their photochemical and photophysical properties [24]. This building block gives the dye good chemical and thermal stability. Due to their large π-conjugated system and high electron-donating nature, julolidine derivatives serve as sensitizers in dye-sensitized solar cells. The bathochrome shift resulting from the replacement of aminophenol units by julolidine assigns these dyes special applicability in several fields of optical imaging and sensing. Lioret et al. described *meso*-pyridyl-substituted *S*- or *Se*-pyronin dyes as type-II photosensitizers (singlet oxygen generation) [23]. Based on the high structure similarity, our newly synthesized compounds **8**–**10** might also act in a similar way. Results concerning the investigation of the mechanism of action will be discussed elsewhere.

## 3. Materials and Methods

### 3.1. Chemistry—Materials and Methods

Melting points (Mp) were determined with a Kofler hot-stage apparatus and are uncorrected. Thin-layer chromatography was performed on silica gel 60 F254; (layer thickness 0.2 mm, (Merck Life Science Kft., an affiliate of Merck KGaA, Darmstadt, Germany)); eluent: a: 10% methanol/90% dichloromethane. The spots were detected with I_2_ or UV (365 nm) after spraying with 5% phosphomolybdic acid in 50% aqueous phosphoric acid and heating at 100–120 °C for 10 min. Flash chromatography was performed on silica gel 60, 40–63 μm (Merck Life Science Kft., an affiliate of Merck KGaA, Darmstadt, Germany). ^1^H NMR spectra were recorded in DMSO-d_6_ or CDCl_3_ solution with a Bruker DRX-500 instrument at 500 MHz. ^13^C NMR spectra were recorded with the same instrument at 125 MHz under the same conditions. Mass spectrometry: full scan mass spectra of the newly synthesized compounds were acquired in the range of 100 to 1100 *m*/*z* with a Q Exactive Plus quadrupole-orbitrap mass spectrometer (Thermo Fisher Scientific, Waltham, MA, USA) equipped with a heated electrospray (HESI). Analyses were performed in positive or negative ion mode by flow injection mass spectrometry with a mobile phase of 50% aqueous acetonitrile containing 0.1 *v*/*v*% formic acid (0.3 mL min^−1^ flow rate). Aliquots of 5 µL of samples were injected into the flow. The ESI capillary was adjusted to 3.5 kV and N_2_ was used as a nebulizer gas.

#### 3.1.1. Synthesis Protocols

##### General One-Pot Procedure for the Synthesis of Rosamines

3-(Diethylamino)phenol (**4Et**) (330 mg, 2 mmol), 3-(dimethylamino)phenol (**4Me**) (274 mg, 2 mmol) or 8-hydroxylulolidine (**4J**) (379 mg, 2 mmol) and the pyridine-2-carbaldehyde (**1**) (107 mg, 1 mmol), 4-bromo-pyridine-2-carbaldehyde (**2**) (185 mg, 1 mmol) or pyridine-4-carbaldehyde (**3**) (107 mg, 1 mmol) and toluene (3 mL) were added into a 10 mL Pyrex pressure vessel (CEM, Part #: 908035) with silicone cap (CEM, Part #: 909210). The mixture was heated in a CEM microwave reactor at 150 °C for 30 min under stirring, and then cooled to room temperature. The residue was dissolved in dichloromethane (10 mL), and cooled to 0 °C. Chloranil (443 mg, 1.80 mmol) dissolved in 1/1 = dichloromethane/methanol mixture (5 mL) was added slowly (during 5 min) to the dichloromethane solution of the residue. The mixture was stirred for 30 min at 0–5 °C. The solvent was evaporated in vacuo, and the crude product was purified by flash chromatography with gradient elution, with dichlorometane to 10% methanol/90% dichloromethane as eluent. The solvent was evaporated in vacuo, and the residue was dissolved in dichloromethane (10 mL) and washed with a saturated aqueous solution of NaCl. After separation of the phases, the aqueous phase was extracted with dichloromethane (2 × 10 mL). The organic phases were combined and dried over Na_2_SO_4_, and the solvent was evaporated in vacuo, giving the chloride salt as a purple solid.

##### Synthesis of Rosamine **8**

As described in Section 3.1.1., starting phenol **4Me** was reacted with aldehyde **1**. Compound **8** was obtained as a purple solid: 220 mg, 64%, Mp.: 277–278 °C, R_f_ = 0.19 ^a^. ^1^H NMR (DMSO-d_6_) δ ppm: 3.29 (s, 12H, 2 × N(CH_3_)_2_); 6.98 (s, 2H, 4-H and 5-H); 7.15 (dd, 2H, *J* = 9.5 Hz, *J* = 2.0 Hz, 2-H and 7-H); 7.27 (d, 2H, *J* = 9.5 Hz, 1-H and 8-H); 7.72 (d, 2H, *J* = 7.7 Hz); 8.14 (t, 1H, *J* = 7.7 Hz); 8.90 (d, 1H, *J* = 4.2 Hz). ^13^C NMR (DMSO-d_6_) δ ppm: 40.4 (2 × N(CH_3_)_2_); 96.3 (2C, 2 × CH); 112.1 (2C, 2 × C); 114.7 (2C, 2 × CH); 124.8 (CH); 125.8 (CH); 130.9 (2C, 2 × CH); 137.2 (CH); 150.1 (CH); 150.6 (C); 153.5 (C); 156.7 (2C, 2 × C); 157.2 (2C, 2 × C). ESI-HRMS: *m*/*z*: 344.17489 [M]^+^ (C_22_H_22_N_3_O^+^ requires 344.17574).

##### Synthesis of Rosamine **9**

As described in Section 3.1.1., starting phenol **4Me** was reacted with aldehyde **2**. Compound **9** was obtained as a purple solid: 279 mg, 66%, Mp.: 215–216 °C, R_f_ = 0.19 ^a^. ^1^H NMR (DMSO-d_6_) δ ppm: 3.30 (s, 12H, 2 × N(CH_3_)_2_); 6.98 (d, 2H, *J* = 2.0 Hz, 4-H and 5-H); 7.14 (dd, 2H, *J* = 9.5 Hz, *J* = 2.0 Hz, 2-H and 7-H); 7.29 (d, 2H, *J* = 9.5 Hz, 1-H and 8-H); 8.01 (dd, 2H, *J* = 5.4 Hz, *J* = 1.6 Hz, 4′-H); 8.07 (d, 1H, *J* = 1.6 Hz, 6′-H); 8.77 (d, 1H, *J* = 5.4 Hz, 3′-H). ^13^C NMR (DMSO-d_6_) δ ppm: 40.4 (2 × N(CH_3_)_2_); 96.3 (2C, 2 × CH); 112.2 (2C, 2 × C); 114.8 (2C, 2 × CH); 127.9 (CH); 128.6 (CH); 130.9 (2C, 2 × CH); 133.0 (C); 151.2 (CH); 152.0 (C); 152.2 (C); 156.8 (2C, 2 × C); 157.1 (2C, 2 × C). ESI-HRMS: *m*/*z*: 422.08602 [M]^+^ (C_22_H_21_BrN_3_O^+^ requires 422.08625).

##### Synthesis of Rosamine **10**

As described in Section 3.1.1., starting phenol **4Me** was reacted with aldehyde **3**. Compound **10** was obtained as a purple solid: 217 mg, 63%, Mp.: 288–289 °C, R_f_ = 0.11 ^a^. ^1^H NMR (DMSO-d_6_) δ ppm: 3.29 (s, 12H, 2 × N(CH_3_)_2_); 6.98 (d, 2H, *J* = 2.0 Hz, 4-H and 5-H); 7.14–7.21 (overlapping multiplets, 4H); 7.57 (d, 2H, *J* = 5.4 Hz); 8.91 (d, 2H, *J* = 4.7 Hz). ^13^C NMR (DMSO-d_6_) δ ppm: 40.5 (2 × N(CH_3_)_2_); 96.3 (2C, 2 × CH); 112.1 (2C, 2 × C); 114.9 (2C, 2 × CH); 123.9 (2C, 2 × CH); 130.7 (2C, 2 × CH); 139.8 (2C, 2 × C); 150.0 (2C, 2 × CH); 153.3 (2C, 2 × C); 156.9 (2C, 2 × C). ESI-HRMS: *m*/*z*: 344.17515 [M]^+^ (C_22_H_22_N_3_O^+^ requires 344.17574).

##### Synthesis of Rosamine **11**

As described in Section 3.1.1., starting phenol **4Et** was reacted with aldehyde **1**. Compound **11** was obtained as a purple solid: 244 mg, 61%, Mp.: 200–202 °C, R_f_ = 0.23 ^a^. ^1^H NMR (DMSO-d_6_) δ ppm: 1.22 (t, *J* = 7.0 Hz, 12H, 2 × N(CH_2_CH_3_)_2_); 3.67 (q, 8H, *J* = 7.0 Hz, 2 × N(CH_2_CH_3_)_2_); 6.99 (d, 2H, *J* = 2.0 Hz, 4-H and 5-H); 7.15 (dd, 2H, *J* = 9.5 Hz, *J* = 2.0 Hz, 2-H and 7-H); 7.27 (d, 2H, *J* = 9.5 Hz, 1-H and 8-H); 7.72 (t, 2H, *J* = 7.4 Hz); 8.14 (t, 1H, *J* = 7.4 Hz); 8.90 (d, 1H, *J* = 4.2 Hz). ^13^C NMR (DMSO-d_6_) δ ppm: 12.3 (2 × N(CH_2_CH_3_)_2_); 45.3 (2 × N(CH_2_CH_3_)_2_); 96.0 (2C, 2 × CH); 112.1 (2C, 2 × C); 114.6 (2C, 2 × CH); 124.8 (CH); 125.8 (CH); 131.2 (2C, 2 × CH); 137.2 (CH); 150.1 (CH); 150.7 (C); 153.1 (C); 155.0 (2C, 2 × C); 157.5 (2C, 2 × C). ESI-HRMS: *m*/*z*: 400.23793 [M]^+^ (C_26_H_30_N_3_O^+^ requires 400.23834).

##### Synthesis of Rosamine **12**

As described in Section 3.1.1., starting phenol **4Et** was reacted with aldehyde **2**. Compound **12** was obtained as a purple solid: 311 mg, 65%, Mp.: 197–19 °C, R_f_ = 0.19 ^a^. ^1^H NMR (DMSO-d_6_) δ ppm: 1.23 (t, *J* = 7.0 Hz, 12H, 2 × N(CH_2_CH_3_)_2_); 3.67 (q, 8H, *J* = 7.0 Hz, 2 × N(CH_2_CH_3_)_2_); 7.00 (d, 2H, *J* = 2.0 Hz, 4-H and 5-H); 7.15 (dd, 2H, *J* = 9.5 Hz, *J* = 2.0 Hz, 2-H and 7-H); 7.27 (d, 2H, *J* = 9.5 Hz, 1-H and 8-H); 8.01 (dd, 2H, *J* = 5.4 Hz, *J* = 1.6 Hz, 4′-H); 8.07 (d, 1H, *J* = 1.6 Hz, 6′-H); 8.77 (d, 1H, *J* = 5.4 Hz, 3′-H). ^13^C NMR (DMSO-d_6_) δ ppm: 12.3 (2 × N(CH_2_CH_3_)_2_); 45.3 (2 × N(CH_2_CH_3_)_2_); 96.1 (2C, 2 × CH); 112.1 (2C, 2 × C); 114.7 (2C, 2 × CH); 127.9 (CH); 128.5 (CH); 131.2 (2C, 2 × CH); 132.9 (C); 151.2 (CH); 151.6 (C); 152.2 (C); 155.1 (2C, 2 × C); 157.4 (2C, 2 × C). ESI-HRMS: *m*/*z*: 478.14816 [M]^+^ (C_26_H_29_BrN_3_O^+^ requires 478.14885).

##### Synthesis of Rosamine **13**

As described in Section 3.1.1., starting phenol **4Et** was reacted with aldehyde **3**. Compound **13** was obtained as a purple solid: 248 mg, 62%, Mp.: 266–269 °C, R_f_ = 0.17 ^a^. ^1^H NMR (DMSO-d_6_) δ ppm: 1.22 (t, *J* = 7.0 Hz, 12H, 2 × N(CH_2_CH_3_)_2_); 3.67 (q, 8H, *J* = 7.0 Hz, 2 × N(CH_2_CH_3_)_2_); 7.00 (d, 2H, *J* = 2.0 Hz, 4-H and 5-H); 7.13–7.20 (overlapping multiplets, 4H); 7.60 (d, 2H, *J* = 5.4 Hz); 8.91 (d, 2H, *J* = 4.7 Hz). ^13^C NMR (DMSO-d_6_) δ ppm: 12.3 (2 × N(CH_2_CH_3_)_2_); 45.3 (2 × N(CH_2_CH_3_)_2_); 96.1 (2C, 2 × CH); 112.0 (2C, 2 × C); 114.7 (2C, 2 × CH); 124.0 (2C, 2 × CH); 131.1 (2C, 2 × CH); 140.3 (C); 149.7 (2C, 2 × C); 152.8 (C); 155.1 (2C, 2 × C); 157.2 (2C, 2 × C). ESI-HRMS: *m*/*z*: 400.23769 [M]^+^ (C_26_H_30_N_3_O^+^ requires 400.23834).

##### Synthesis of Rosamine **14**

As described in Section 3.1.1., starting phenol **4J** was reacted with aldehyde **1**. Compound **14** was obtained as a purple solid: 282 mg, 63%, Mp.: 218–219 °C, R_f_ = 0.23 ^a^. ^1^H NMR (CDCl_3_) δ ppm: 1.95 (m, 4H); 2.08 (m, 4H); 2.67 (m, 4H); 3.00 (m, 4H); 3.51–3.56 (overlapping multiplets, 8H); 6.72 (s, 2H); 7.46 (d, 1H, *J* = 7.7 Hz); 7.55 (dd, 1H, *J* = 7.7 Hz, *J* = 4.7 Hz); 8.03 (dt, *J* = 7.7 Hz, *J* = 1.5 Hz); 8.82 (d, 1H, *J* = 4.7 Hz). ^13^C NMR (CDCl_3_) δ ppm: 19.6 (2C, 2 × CH_2_); 19.8 (2C, 2 × CH_2_); 20.5 (2C, 2 × CH_2_); 27.6 (2C, 2 × CH_2_); 50.5 (2C, 2 × CH_2_); 51.0 (2C, 2 × CH_2_); 105.4 (2C, 2 × C); 112.3 (2C, 2 × C); 123.9 (2C, 2 × C); 124.4 (CH); 125.7 (CH); 126.1 (2C, 2 × CH); 137.2 (CH); 150.1 (CH); 151.1 (C); 151.2 (2C, 2 × C); 151.8 (C); 152.4 (2C, 2 × C). ESI-HRMS: *m*/*z*: 448.23836 [M]^+^ (C_30_H_30_N_3_O^+^ requires 448.23834).

##### Synthesis of Rosamine **15**

As described in Section 3.1.1., starting phenol **4J** was reacted with aldehyde **2**. Compound **15** was obtained as a purple solid: 316 mg, 60%, Mp.: 295–296 °C, R_f_ = 0.26 ^a^. ^1^H NMR (DMSO-d_6_) δ ppm: 1.87 (m, 4H); 2.00 (m, 4H); 2.69 (m, 4H); 2.98 (m, 4H); 3.51–3.54 (overlapping multiplets, 8H); 6.74 (s, 2H); 7.92 (d, 1H, *J* = 1.6 Hz); 7.96 (dd, 1H, *J* = 5.4 Hz, *J* = 1.6 Hz); 8.72 (d, 1H, *J* = 5.4 Hz). ^13^C NMR (DMSO-d_6_) δ ppm: 19.0 (2C, 2 × CH_2_); 19.2 (2C, 2 × CH_2_); 20.0 (2C, 2 × CH_2_); 26.7 (2C, 2 × CH_2_); 49.7 (2C, 2 × CH_2_); 50.3 (2C, 2 × CH_2_); 105.0 (2C, 2 × C); 111.4 (2C, 2 × C); 123.9 (2C, 2 × C); 125.4 (2C, 2 × CH); 127.5 (CH); 128.1 (CH); 129.4 (CH); 132.9 (C); 149.1 (C); 150.6 (2C, 2 × C); 151.1 (CH); 151.4 (C); 153.0 (C). ESI-HRMS: *m*/*z*: 526.14883 [M]^+^ (C_30_H_29_BrN_3_O^+^ requires 526.14885).

##### Synthesis of Rosamine **16**

As described in Section 3.1.1., starting phenol **4J** was reacted with aldehyde **3**. Compound **16** was obtained as a purple solid: 273 mg, 61%, Mp.: 204–206 °C, R_f_ = 0.17 ^a^. ^1^H NMR (DMSO-d_6_) δ ppm: 1.86 (m, 4H); 2.00 (m, 4H); 2.68 (m, 4H); 2.98 (m, 4H); 3.50–3.55 (overlapping multiplets, 8H); 6.72 (s, 2H); 7.45 (d, 2H, *J* = 5.3 Hz); 8.85 (d, 2H, *J* = 5.3 Hz). ^13^C NMR (DMSO-d_6_) δ ppm: 19.0 (2C, 2 × CH_2_); 19.2 (2C, 2 × CH_2_); 19.9 (2C, 2 × CH_2_); 26.7 (2C, 2 × CH_2_); 49.7 (2C, 2 × CH_2_); 50.2 (2C, 2 × CH_2_); 105.0 (2C, 2 × C); 111.1 (2C, 2 × C); 123.9 (2C, 2 × C); 124.0 (2C, 2 × CH); 125.3 (2C, 2 × CH); 140.5 (C); 149.9 (2C, 2 × CH); 150.0 (C); 150.7 (2C, 2 × C); 151.1 (2C, 2 × C). ESI-HRMS: *m*/*z*: 448.23775 [M]^+^ (C_30_H_30_N_3_O^+^ requires 448.23834).

### 3.2. Spectroscopic Measurements

Stock solutions of **8**–**16** were prepared in DMSO (10 mM each). Thereafter, samples were further diluted in phosphate-buffered saline (PBS, pH 7.4). Absorption and fluorescence spectra were recorded applying a Hitachi U-3900 UV-Vis spectrophotometer (Tokyo, Japan) and a Hitachi F-4500 (Tokyo, Japan) fluorometer at 25 °C, respectively.

A fluorescence spectrofluorometer (HORIBA Jobin-Yvon, NanoLog FL3-2iHR, Paris, France) with an integrating sphere accessory (HORIBA Quanta-phi F-3029) was used to measure and calculate the fluorescence quantum yield. They were connected through an optical fiber interface. The light source was a 450 W xenon lamp. NanoLog works using single photon counting, which ensures very high sensitivity. Quanta–phi is a quantum yield and CIE measurement accessory for NanoLog spectrofluorometers. It features a large 150 mm integrating sphere. Standard 12 mm quartz cuvettes were used into its top-loading, center-mounted liquid sample holder. For each quantum yield calculation, four spectra were measured with 0.1 nm spectral steps: (1) sample fluorescence, (2) sample Rayleigh-scattering, (3) blank fluorescence and (4) blank Rayleigh-scattering. All the calculations were made using the NanoLog’s QY/colorimetry software: FluorEssence™ for Windows^®^.

FluorEsscence version number: 3.9.0.1 Origin version: 8.6001. The spectrofluorometer’s built-in spectral sphere correction file was applied for the precise calculation.

### 3.3. Phototoxicity Measurements

A431 epidermoid carcinoma cell line (ATCC) was used for toxicity measurements. The cells were cultured in DMEM (Gibco, Thermo Fischer Scientific (Waltham, MA, USA)) supplemented with 10% fetal calf serum, 100 U ml^−1^ penicillin, 100 μg ml^−1^ streptomycin and 2 mM l-glutamine at 37 °C in a humidified incubator with an atmosphere of 5% CO_2_ with 5% CO_2._ Pyridyl rosamines were dissolved in DMSO to obtain a stock solution of 20 mM and diluted to their required concentration in culture medium.

For light-induced cytotoxicity measurements, the cells were seeded at a density of 7.5 × 10^3^/well in 48-well plates (ThermoScientific) and incubated for 24 h. The next day, the rosamines stock solutions (20 mM in DMSO) were diluted with medium and the cells were treated with increasing concentrations of different rosamines for 24 h. The medium was then removed from the cells, and fresh culture medium was added to each well. Then, the cells were exposed to an LED light irradiance of 4 W cm^−2^ at λ = 605 nm (**14**–**16**) or λ = 550 nm (**8**–**13**) with periodical 30/15/30 s illumination with in-house equipment and further incubated for 48 h at 37 °C. Cell viability was determined using the PrestoBlue reagent (ThermoFisher Scientific, Waltham, MA, USA) according to the manufacturer’s instructions. Briefly, after 60 min of incubation with 5% Prestoblue reagent at 37 °C, the fluorescence of the cells was determined using an Enspire plate reader, at Ex/Em 555/585 nm. As for dark toxicity of each photosensitizer, cell viability was evaluated as described above except that no illumination was provided. Cells treated only with cell culture medium served as controls for cell viability (untreated cells) either on the irradiated plates or on plates that were kept in the dark. Cell viability (%) was calculated by comparing the fluorescence of untreated and rosamine-treated cells. Data represent the average of three independent experiments. Average  ±  SD values are shown. IC_50_ values were calculated by GraphPad Prism 6 software (GraphPad, La Jolla, CA, USA).

## 4. Conclusions

A nine-member pyridyl rosamine compound library has been synthesized via aldehyde–pyrrole condensation strategy. An effective microwave-assisted condensation methodology was elaborated without utilizing any catalyst or additive. The oxidation process of triarylmethane intermediates has been renewed by slow addition of a 10% chloranil in solution of dichloromethane/methanol (1/1) at 0–5 °C. The photophysical characterization of the dyes was performed by investigation of their UV-Vis absorption and fluorescence emission spectra in DMSO solvent or PBS buffer. Absolute fluorescence quantum yields were measured for two compounds. The applicability of the pyridyl rosamines (**8**–**16**) as PSs was investigated against the epidermoid carcinoma cell line A431. The compounds displayed light-induced toxicity in the submicromolar or occasionally in the low nanomolar range. Concerning the efficacy of PD, julolidine derivatives **14**–**16** should be highlighted with their PI data near or above 50. The *meso*-(4-pyridyl) julolidine-based derivative **16** stands out from the test compounds in two ways. Namely, it provided the highest light-induced toxicity and it also showed an excellent PI above 100, indicating a two orders of magnitude increase in light-induced cytotoxic efficacy in addition to its dark toxicity. Interestingly, the PI value of 5.5 for compound **9** is far below those of the others. Since the rosamines (**8**–**16**) possess high fluorogenicity in the orange spectral range, this early study highlights *meso*-pyridyl-substituted xanthene dyes as promising phototheranostic candidates suitable for combined fluorescence diagnosis and PDT. The present research could serve as a starting point for a more detailed, in-depth study covering a broad panel of cancer and normal cell lines. After a thorough study of efficiency and selectivity, the most promising phototheranostics could even reach the in vivo assessment.

## Figures and Tables

**Table 1:** Effect of the reaction conditions on the oxidation of C-9 of compound **5**.

Entry	Oxidizing Agent ^a^	Reaction Temperature (°C)	Reaction Time (Min)	Yield (%)
1	1,4-benzoquinone ^b^	rt	60	-
2	DDQ ^b^	rt	30	11%
3	DDQ ^c^	rt	30	25%
4	DDQ ^c^	0–5	30	57%
5	chloranil ^b^	rt	30	20%
6	chloranil ^c^	rt	30	39%
7	chloranil ^c^	0–5	30	78%

^a^ added in 1.8 equiv. ^b^ added as solid in one portion. ^c^ added in 10% solution in dichloromethane/methanol = 1/1 (over 5 min).

**Table 2:** UV-Vis and fluorescence spectroscopic properties of **8**–**16** in phosphate-buffered saline (PBS; λ_abs_, absorption wavelength maximum; λ_em_, fluorescence emission wavelength maximum; Δλ, stokes shift; ε_max_, maximum molar absorptivity).

Rosamine Compound	Solvent	λ_abs_ [nm]	λ_em_ [nm]	Δλ [nm]	ε_max_ [M^−1^ cm^−1^]
**8**	PBS (pH 7.4)	564	587	23	9.15 × 10^4^
**9**	PBS (pH 7.4)	568	593	25	6.08 × 10^4^
**10**	PBS (pH 7.4)	559	583	24	4.55 × 10^4^
**11**	PBS (pH 7.4)	571	593	22	10.15 × 10^4^
**12**	PBS (pH 7.4)	574	601	27	10.03 × 10^4^
**13**	PBS (pH 7.4)	567	592	25	8.43 × 10^4^
**14**	PBS (pH 7.4)	588	610	22	4.25 × 10^4^
**15**	PBS (pH 7.4)	594	617	23	6.58 × 10^4^
**16**	PBS (pH 7.4)	587	611	24	7.10 × 10^4^

**Table 3:** IC_50_ values of pyridyl rosamines in A431 cells kept in the dark and after irradiation and the corresponding phototoxic index (PI) values.

Pyridyl Rosamines	IC_50_ (μM) Dark	IC_50_ (μM) Light-Induced	PI
**8**	2.449 ± 0.601	0.200 ± 0.048	12.3
**9**	0.578 ± 0.091	0.106 ± 0.037	5.5
**10**	14.383 ± 3.532	0.572 ± 0.140	25.1
**11**	0.730 ± 0.150	0.053 ± 0.009	13.9
**12**	0.257 ± 0.062	0.020 ± 0.004	13.1
**13**	3.082 ± 0.450	0.163 ± 0.049	18.9
**14**	0.448 ± 0.057	0.009 ± 0.003	51.7
**15**	0.473 ± 0.035	0.010 ± 0.001	47.2
**16**	0.623 ± 0.101	0.005 ± 0.001	127.3

## Data Availability

Data are available upon request.

## Supplementary Materials

The following supporting information can be downloaded at: https://www.mdpi.com/article/10.3390/ijms26041482/s1.
