# Supplementary material for: Facile Synthesis of Pyridyl Rosamines as Potential Photosensitizers
Source: Int J Mol Sci. 2025 Feb 10;26(4):1482. doi: 10.3390/ijms26041482 (PMC11855136; doi:10.3390/ijms26041482)

# Facile Synthesis of Pyridyl Rosamines as Potential Photosensitizers

**Éva Bakos[1], Henrietta Ágoston[2], Rebeka Ignácz[2,3], Attila Hunyadi[3,9], Miklós Poór[4,5], János Erostyák[6,7], Zoltán Kele[8], Csilla Özvegy-Laczka[1], Erzsébet Mernyák[2,3*]**

[1] Drug resistance research group, Institute of Enzymology, Research Centre for Natural Sciences, HUN-REN, Magyar tudósok körútja 2, H-1117 Budapest, Hungary; bakos.eva@ttk.mta.hu, laczka.csilla@ttk.hu
[2] Department of Molecular and Analytical Chemistry, University of Szeged, Dóm tér 8, H-6720 Szeged, Hungary; hencsike12@gmail.com, ignaczrebi2000@gmail.com, mernyak.erzsebet@szte.hu
[3] Department of Pharmacognosy, University of Szeged, Eötvös u 6, H-6720 Szeged, Hungary; ignaczrebi2000@gmail.com, hunyadi.attila@szte.hu, mernyak.erzsebet@szte.hu
[4] Department of Laboratory Medicine, Medical School, University of Pécs, Ifjúság útja 13, Pécs H-7624, Hungary; poor.miklos@pte.hu
[5] Molecular Medicine Research Group, János Szentágothai Research Centre, University of Pécs, Ifjúság útja 20, Pécs H-7624, Hungary; poor.miklos@pte.hu
[6] Department of Experimental Physics, Faculty of Sciences, University of Pécs, Ifjúság útja 6, Pécs H-7624, Hungary; erostyak@fizika.ttk.pte.hu
[7] Molecular Biophysics Research Group, János Szentágothai Research Centre, University of Pécs, Ifjúság útja 20, Pécs H-7624, Hungary; erostyak@fizika.ttk.pte.hu
[8] Department of Medicinal Chemistry, University of Szeged, Dóm tér 8, H-6720 Szeged, Hungary; kele.zoltan@med.u-szeged.hu
[9] HUN-REN-SZTE Biologically Active Natural Products Research Group, Eötvös u. 6, H-6720 Szeged, Hungary; hunyadi.attila@szte.hu.

* Correspondence: mernyak.erzsebet@szte.hu

Academic Editor: Firstname Lastname

Received: 20 December 2024
Revised: 2 February 2025
Accepted: 5 February 2025
Published: 10 February 2025

**Citation:** To be added by editorial staff during production.

**Copyright:** © 2025 by the authors. Submitted for possible open access publication under the terms and conditions of the Creative Commons Attribution (CC BY) license (https://creativecommons.org/licenses/by/4.0/).

$^{1}$H and $^{13}$C NMR spectra of the compounds

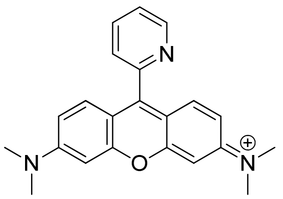

Chemical Formula: $C_{22}H_{22}N_3O^+$
Exact Mass: 344,18

**8**

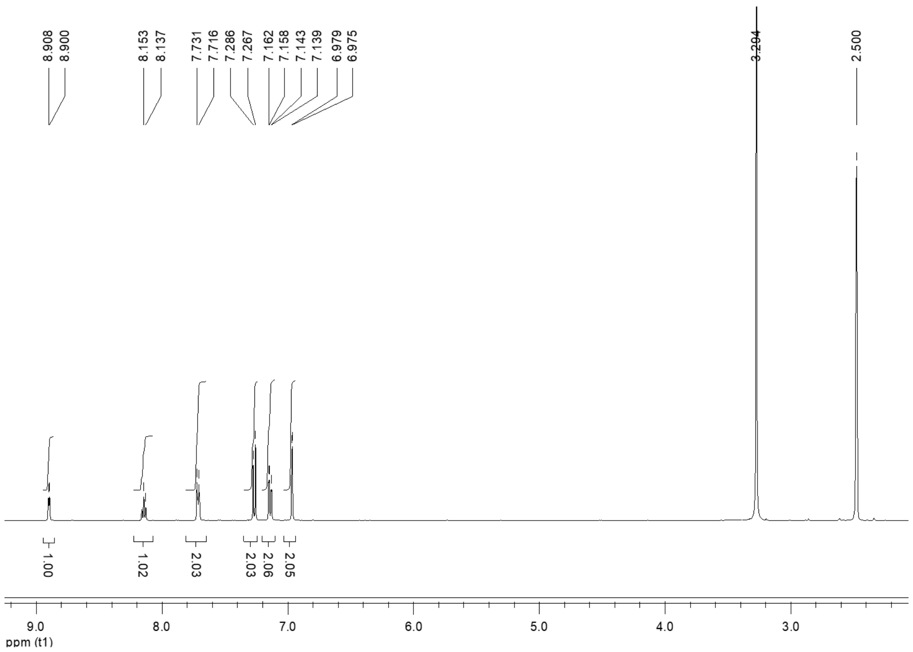


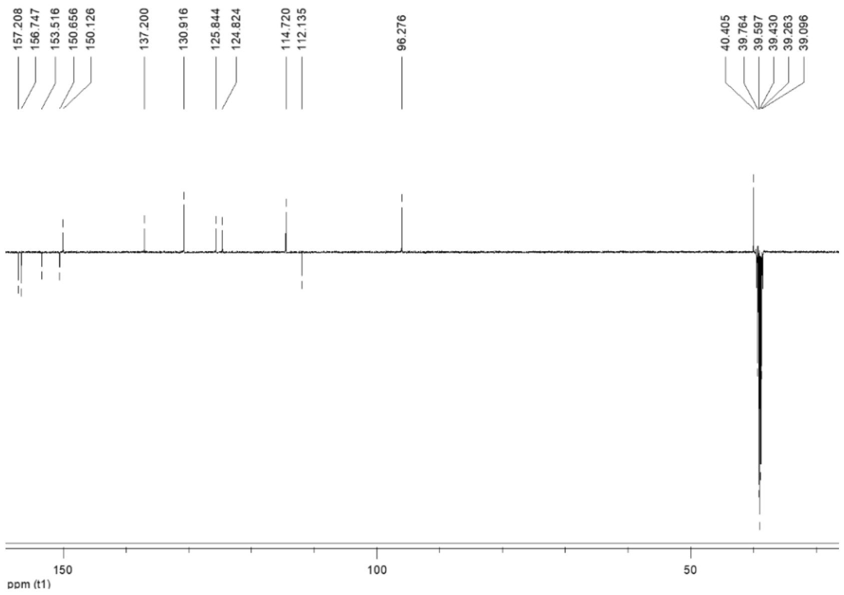

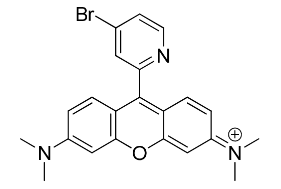


Chemical Formula: $C_{22}H_{21}BrN_3O^+$
Exact Mass: 422,09

**9**

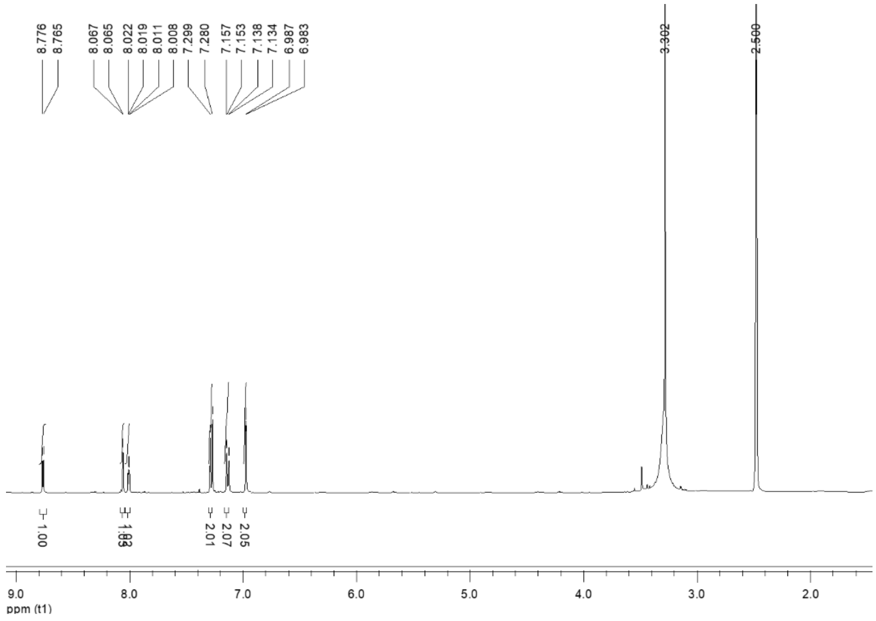


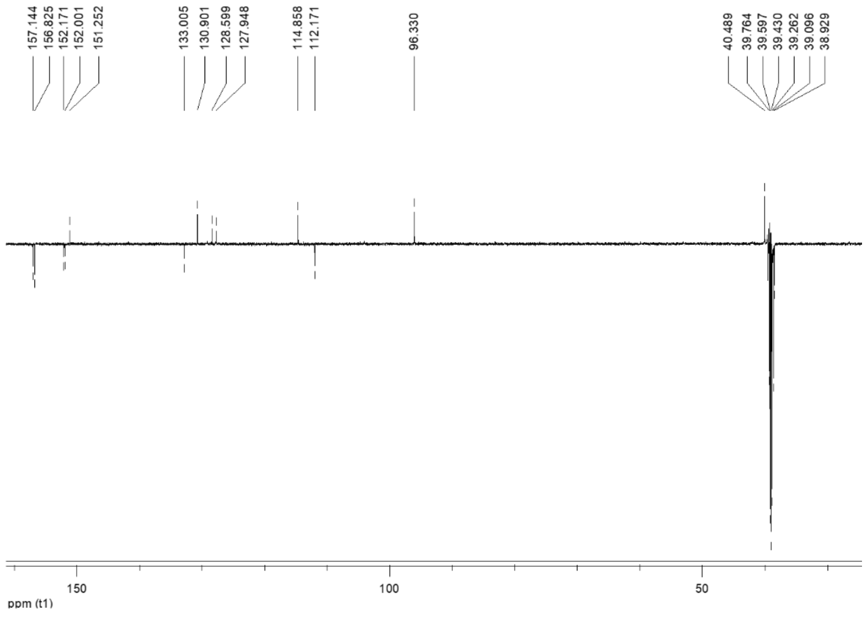

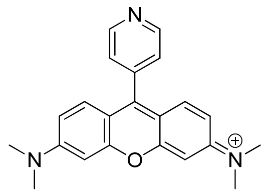

Chemical Formula: $C_{22}H_{22}N_3O^+$
Exact Mass: 344,18

**10**

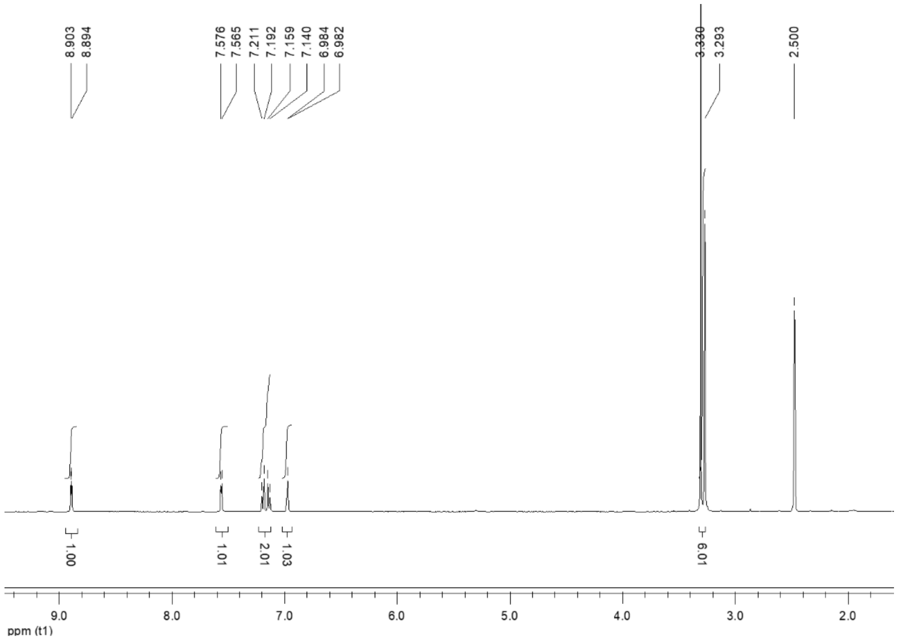


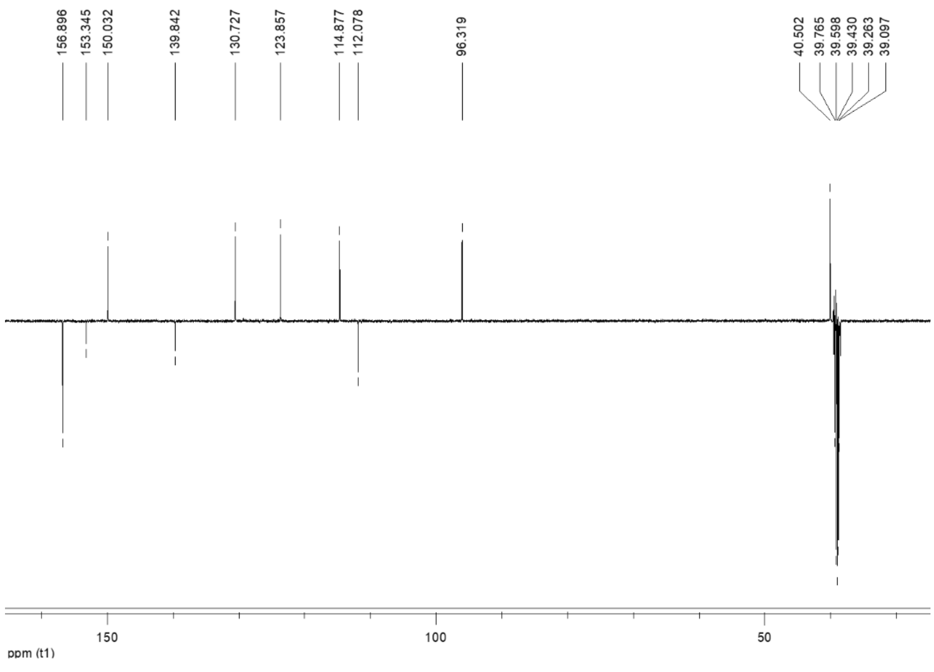

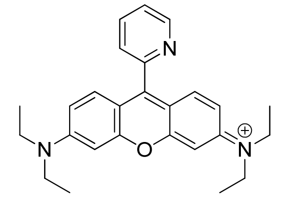


Chemical Formula: $C_{26}H_{30}N_3O^+$
Exact Mass: 400,24

**11**

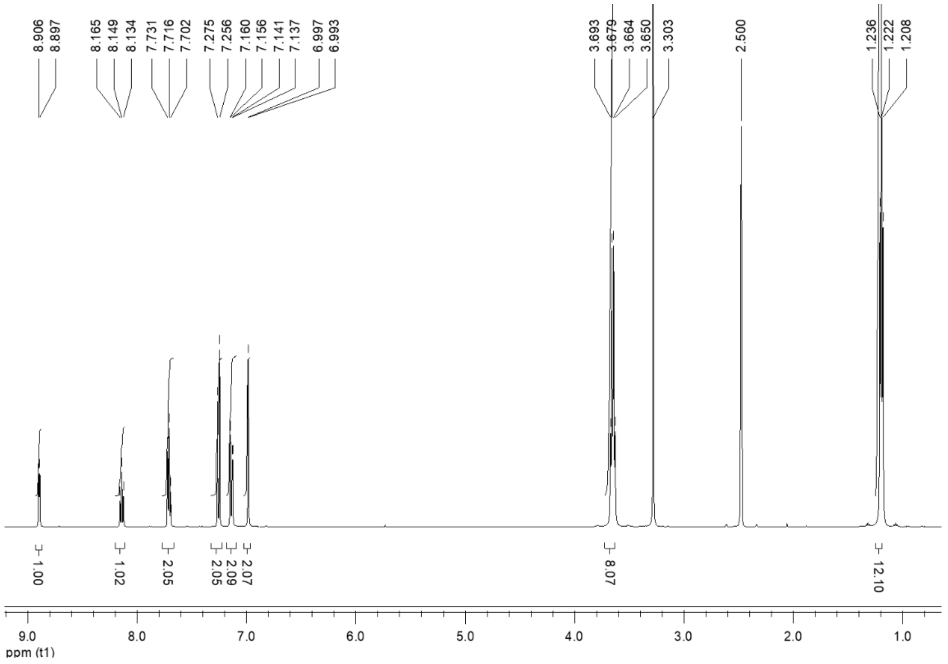


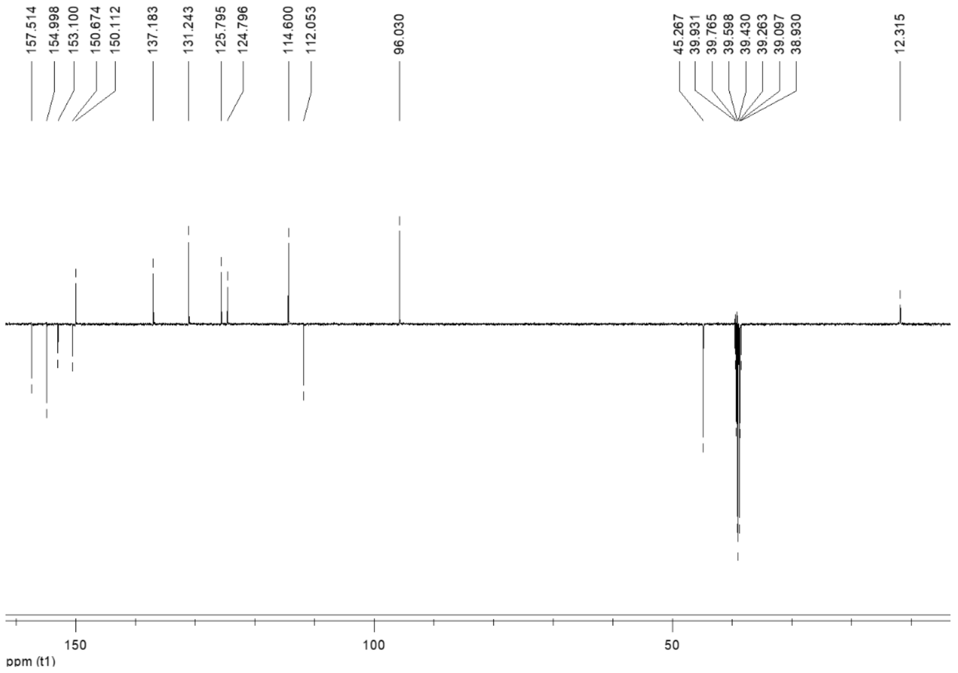

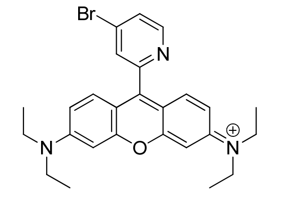


Chemical Formula: $C_{26}H_{29}BrN_3O^+$
Exact Mass: 478,15

**12**

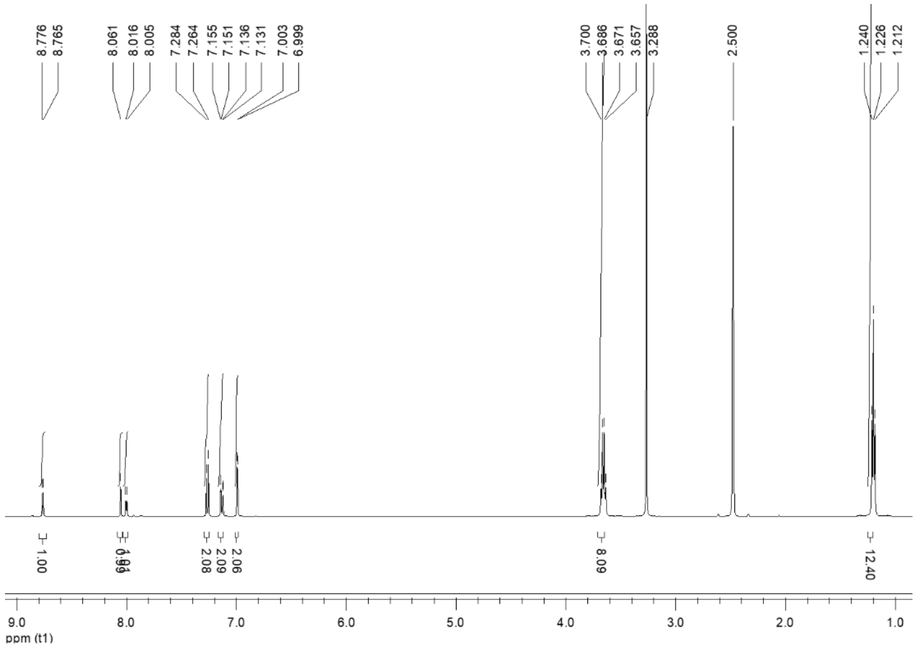


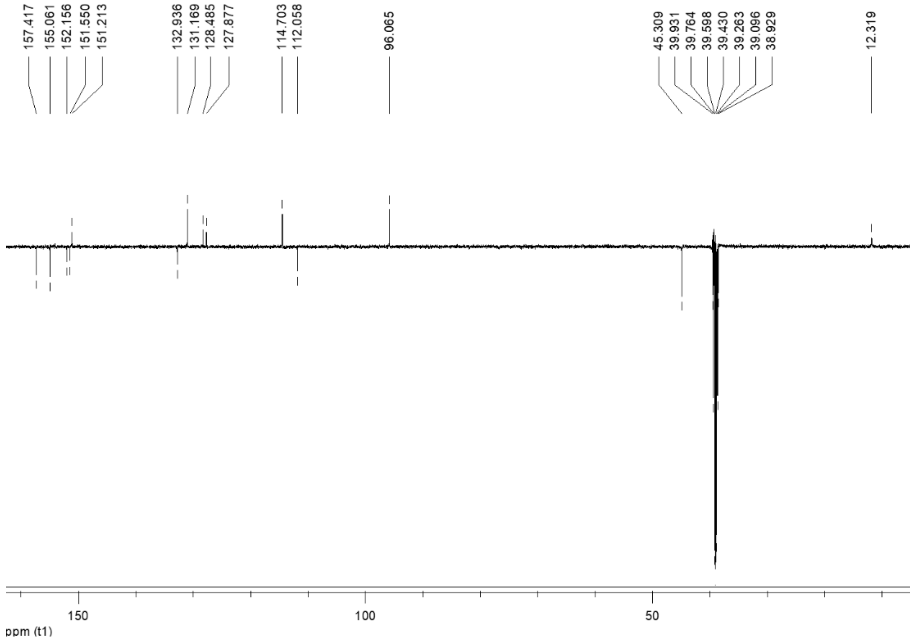

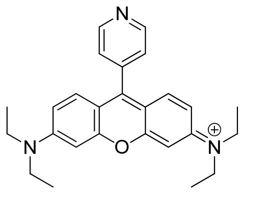


Chemical Formula: $C_{26}H_{30}N_3O^+$
Exact Mass: 400,24

**13**

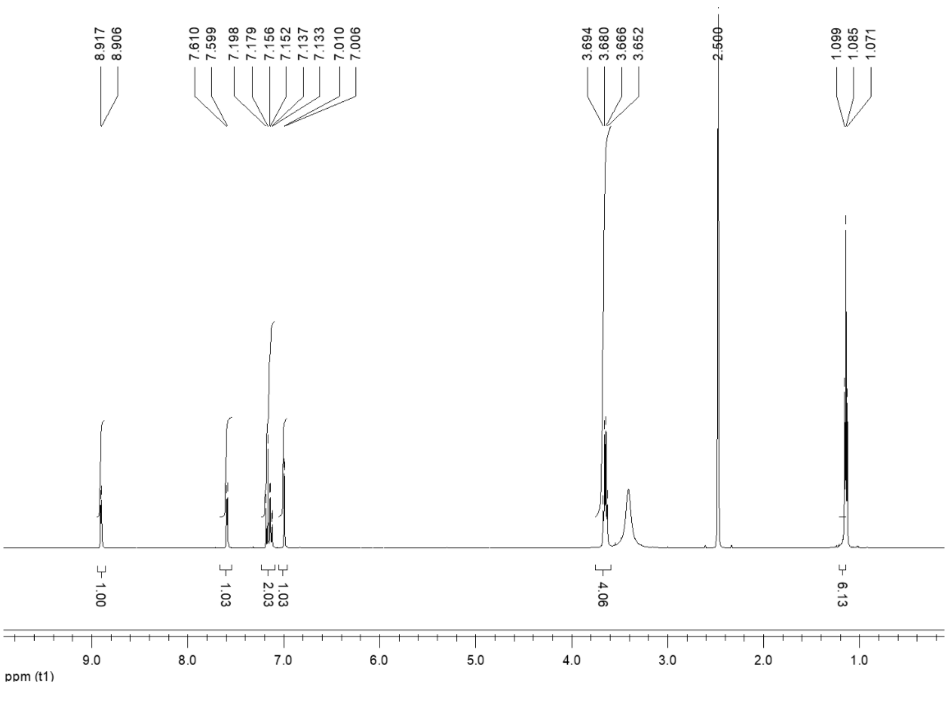


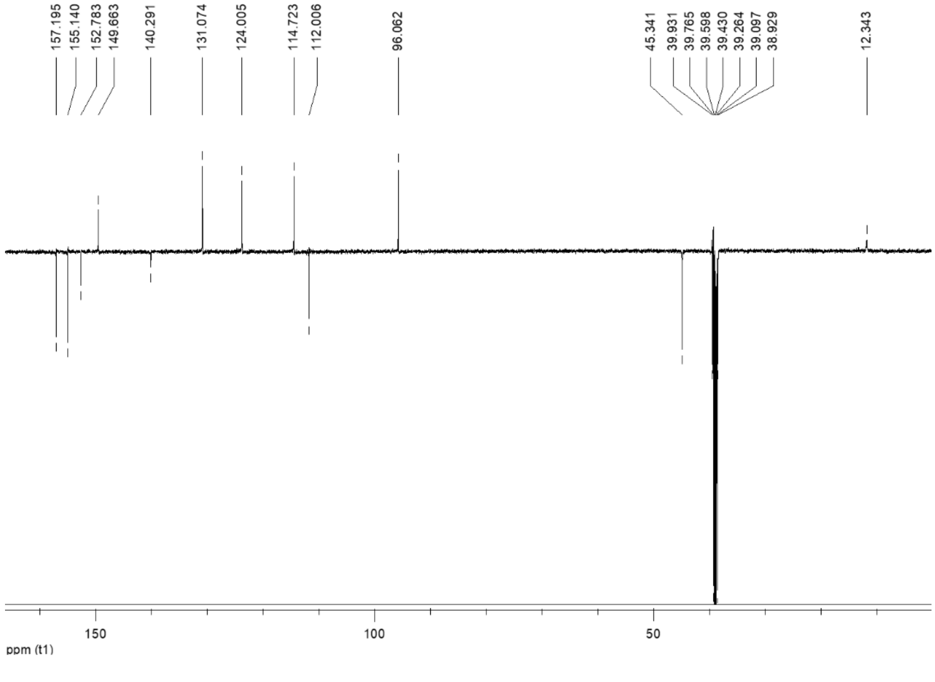

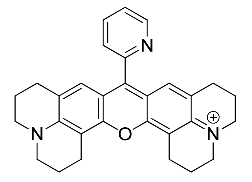


Chemical Formula: $C_{30}H_{30}N_3O^+$
Exact Mass: 448,24

**14**

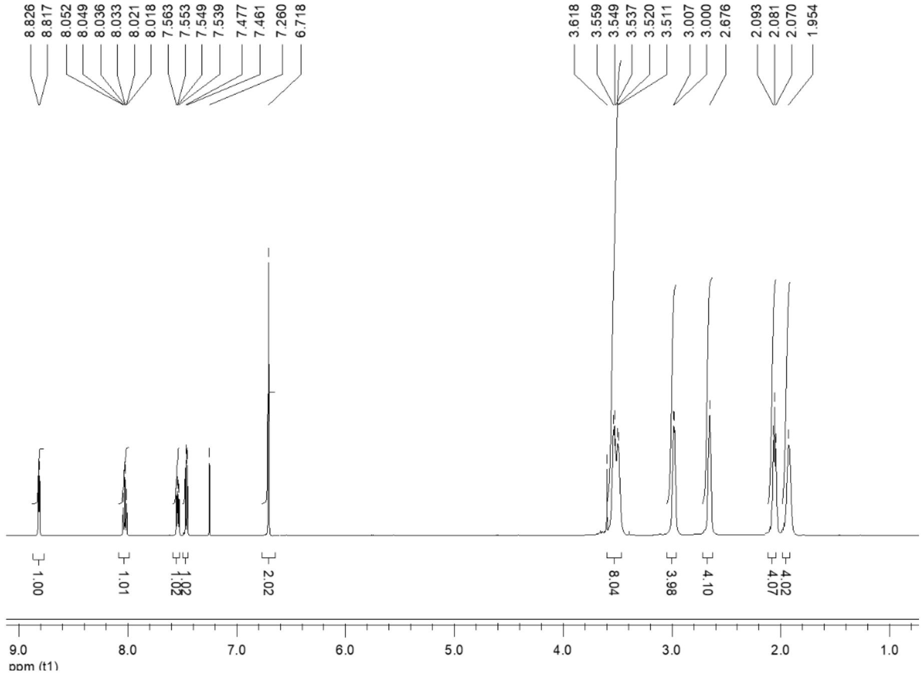


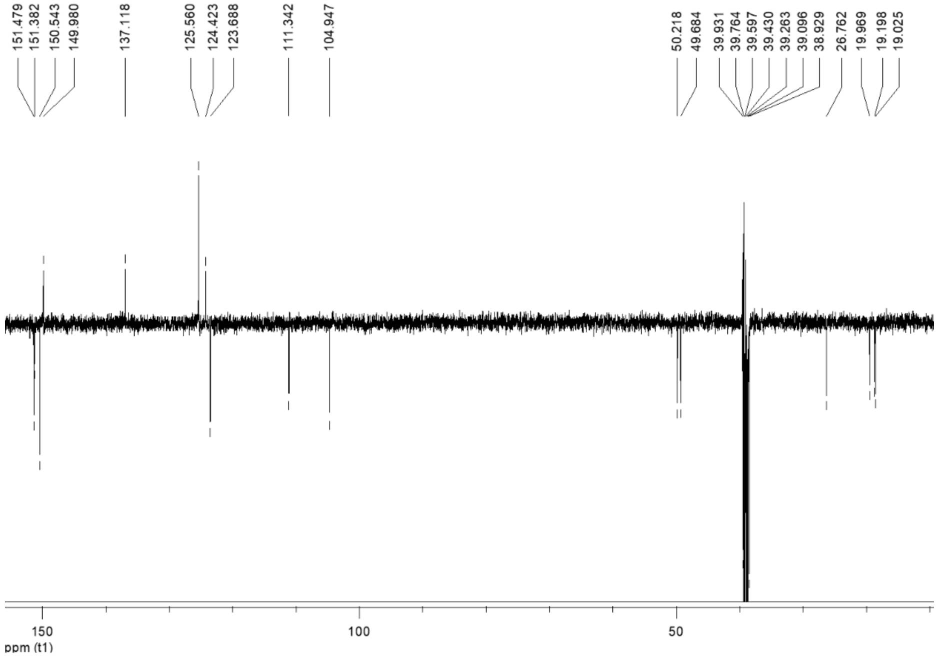

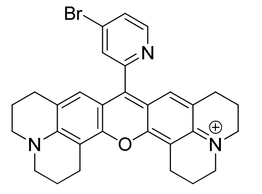


Chemical Formula: $C_{30}H_{29}BrN_3O^+$
Exact Mass: 526,15

**15**

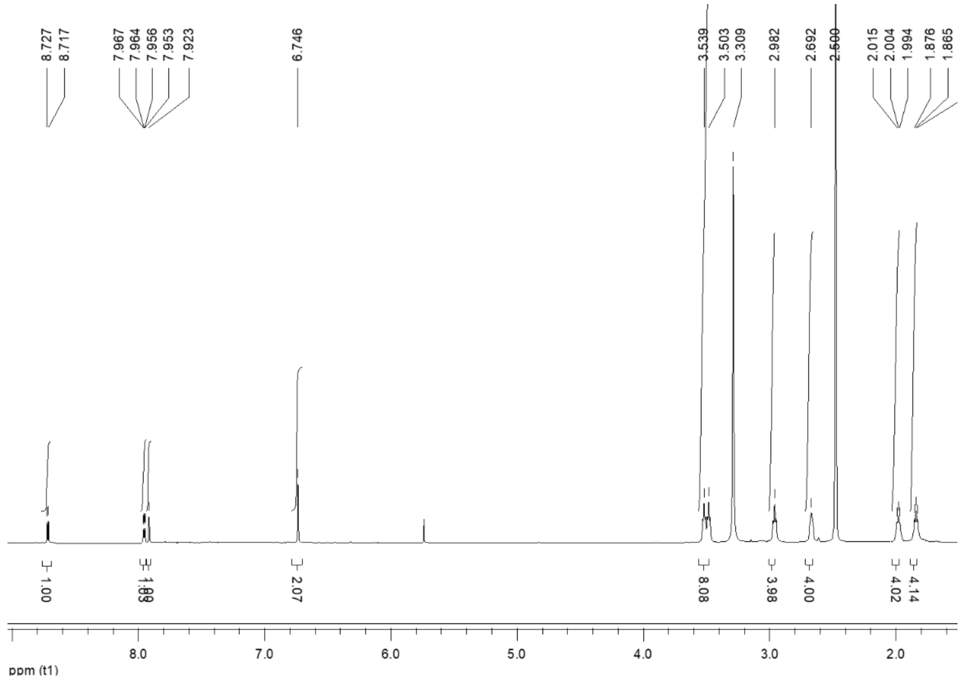


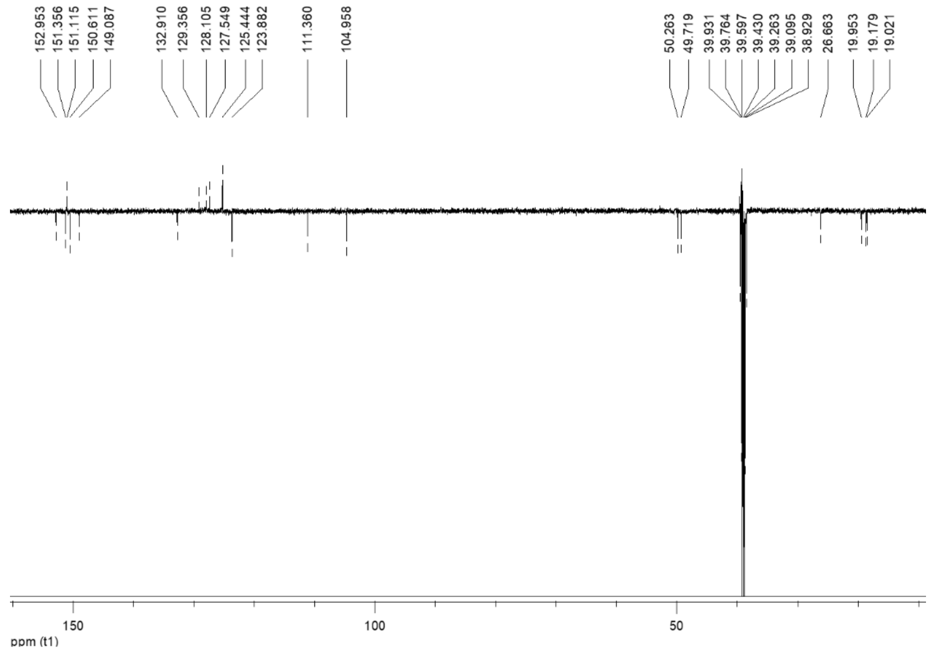

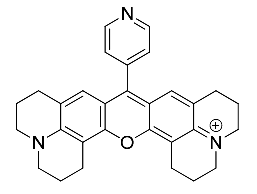


Chemical Formula: $C_{30}H_{30}N_3O^+$
Exact Mass: 448,24

**16**

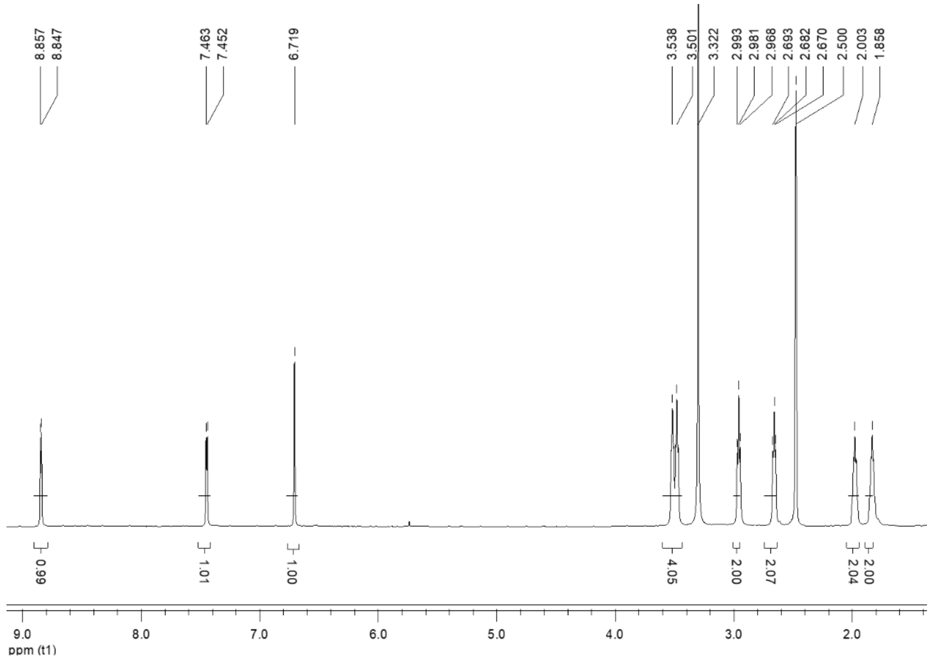


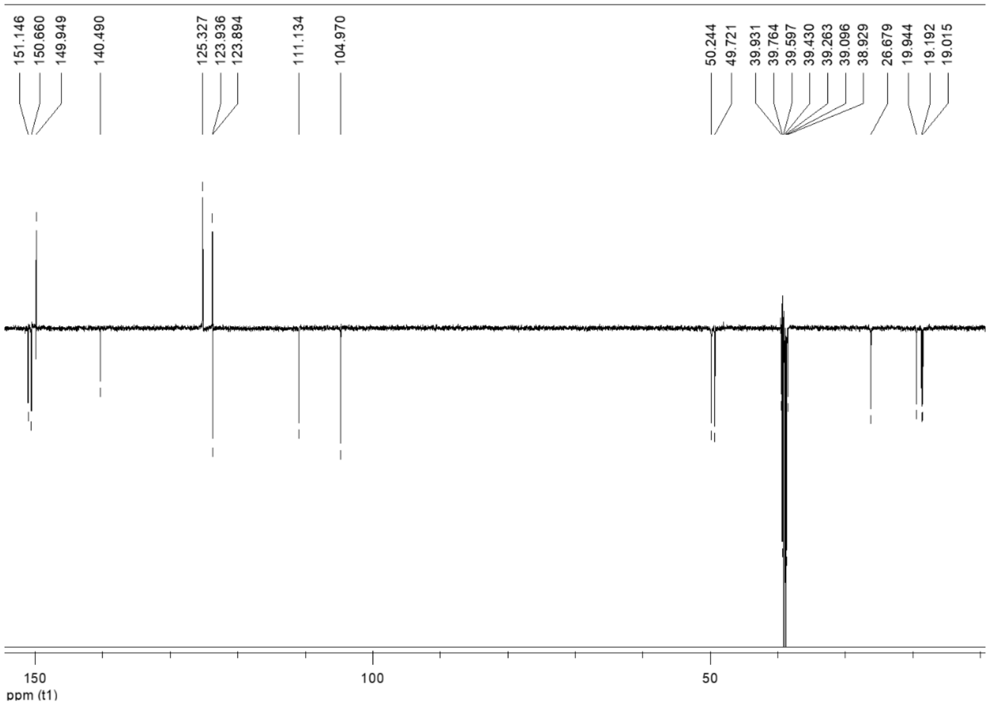

Supplement: Supplementary file 1 [file ijms-26-01482-s001.zip › ijms-3411477-supplementary.pdf]
